# Ultrasound and Nanomedicine for Cancer-Targeted Drug Delivery: Screening, Cellular Mechanisms and Therapeutic Opportunities

**DOI:** 10.3390/pharmaceutics14061282

**Published:** 2022-06-16

**Authors:** Chien-Hsiu Li, Yu-Chan Chang, Michael Hsiao, Ming-Hsien Chan

**Affiliations:** 1 Genomics Research Center, Academia Sinica, Taipei 115, Taiwan; g803020178g@gate.sinica.edu.tw; 2 Department of Biomedical Imaging and Radiological Sciences, National Yang Ming Chiao Tung University, Taipei 112, Taiwan; yuchanchang@ym.edu.tw; 3 Department of Biochemistry, College of Medicine, Kaohsiung Medical University, Kaohsiung 807, Taiwan

**Keywords:** ultrasound, nanomedicine, drug screening, cellular mechanisms, therapeutic drug delivery system

## Abstract

Cancer is a disease characterized by abnormal cell growth. According to a report published by the World Health Organization (WHO), cancer is the second leading cause of death globally, responsible for an estimated 9.6 million deaths in 2018. It should be noted that ultrasound is already widely used as a diagnostic procedure for detecting tumorigenesis. In addition, ultrasound energy can also be utilized effectively for treating cancer. By filling the interior of lipospheres with gas molecules, these particles can serve both as contrast agents for ultrasonic imaging and as delivery systems for drugs such as microbubbles and nanobubbles. Therefore, this review aims to describe the nanoparticle-assisted drug delivery system and how it can enhance image analysis and biomedicine. The formation characteristics of nanoparticles indicate that they will accumulate at the tumor site upon ultrasonic imaging, in accordance with their modification characteristics. As a result of changing the accumulation of materials, it is possible to examine the results by comparing images of other tumor cell lines. It is also possible to investigate ultrasound images for evidence of cellular effects. In combination with a precision ultrasound imaging system, drug-carrying lipospheres can precisely track tumor tissue and deliver drugs to tumor cells to enhance the ability of this nanocomposite to treat cancer.

## 1. Introduction

A primary means of diagnosing diseases, including cancer, is through liquid or tissue biopsies and imaging tests. Generally, preliminary testing is accomplished via collection (e.g., blood, urine) and other noninvasive methods [1,2]. It is usually possible to visualize deep tissue through contrast imaging methods such as X-rays [3], computed tomography (CT) [4], magnetic resonance imaging (MRI) [5], positron emission tomography (PET) scan [6], radionuclide scan, or ultrasound [7]. To visualize these images, contrast agents are injected into the tissue. The injected contrast agent makes a difference in the distribution of signals throughout the tissue, which is used as a basis for the interpretation of the lesion. Nevertheless, some imaging agents are toxic and can cause side effects in some people [8]. In recent years, nanomaterials have reduced side effects [9]. Ultrasound has come to be regarded as a more accurate detection method that is free of radiation concerns, such as for detecting internal organs or for use on pregnant women, with the advancement of image resolution [10]. The vibration range that the human ear can hear is about 15 to 20,000 Hz. Ultrasound therapy equipment drives high-frequency vibrations (higher than 20,000 Hz) into a patient’s body and pours energy into it to achieve therapeutic effects. There are several types of vibration: A continuous wave means that it vibrates all the time until turned off [11]. Another is two milliseconds of on–off switch wave, followed by two milliseconds of rest; this kind of vibration is referred to as 1:1 due to the vibration time being the same as the rest time, meaning that it is only operating 50% of the time [12]. Similar to the above, but in different proportions, others use ratios such 1:2 (33%), 1:3 (25%), 1:4 (20%) for vibration and rest time. Different vibration intervals depend on what kind of energy needs to be transmitted. Is it a little more thermal energy? Or a little more vibration? It is also possible to choose whether the interval is two or four milliseconds. In addition to frequency, doctors can also decide how much energy to send into the patient’s body [13]. If they want to use heat-based ultrasonic therapy, it may be a continuous vibration coupled with a 0.5 W/(cm^2^) parameter. The doctors can manipulate 0.5 joules of energy per second per square centimeter of the area [14].

The ultrasonic phased array method was developed to accurately quantify the use of ultrasonic energy and calculate the information intensity and imaging time for ultrasonic imaging. The ultrasonic imaging technique can be analyzed with respect to each emitted array element with ultrasonic phased array detection to realize beam angle adjustment, depth of focus variation, and electron scan. The evaluation of ultrasonic phased array imaging methods, based on the ultrasonic algorithm, has been intensively studied by scholars. The concept of full-matrix capture (FMC) was first proposed by Holmes et al., and a full-focus imaging method (TFM) based on FMC was established [15]. Compared to the conventional ultrasonic phased array focusing algorithm, TFM can achieve synthetic focusing on any point in the detection area with significantly better imaging quality. Therefore, the calculation method is widely used in the fields of aviation, nuclear power, and human organs. Image reconstruction using FMC is divided into two steps. First, the examination area is discretized into a grid. Second, the amplitude intensity at the grid point $P\left(x_{p},z_{p}\right)$ is calculated with the following equation:

(1) $$I_{p}\left(x_{p},z_{p}\right)=\overset{n}{\underset{i=1}{\sum }}\overset{n}{\underset{j=1}{\sum }}A_{ij}\left(t_{ij}\left(x_{p},z_{p}\right)\right)$$

where $t_{ij}\left(x_{p},z_{p}\right)$ is the time of flight from the transmitting element, i, through the focus point, $P\left(x_{p},z_{p}\right)$, to the receiving element, j. The time of flight is defined as:

(2) $$t_{ij}\left(x_{p},z_{p}\right)=\frac{\left(\sqrt{{\left(x_{i}-x_{p}\right)}^{2}+z_{p}^{2}}+\sqrt{{\left(x_{j}-x_{p}\right)}^{2}+z_{p}^{2}}\right)}{c}$$

where c is the propagation velocity of ultrasound in the medium and $\left(x_{i},0\right)$ and $\left(x_{j},0\right)$ are the Cartesian coordinates of the transmitter and receiver, respectively.

Basically, the body needs to be able to absorb these energies in order for them to have a healing effect. Different tissues in the body have different abilities to absorb ultrasonic energy. The energy is converted into heat and causes tissue temperature to rise. Therefore, ultrasound can be used as a deep heat compress [16]. Under the correct operation conditions, it can cause tissue to warm up to 40–45 degrees Celsius, and the tissue will be congested, promoting circulation. Ultrasound creates tiny eddies in the body called sonic flow [17,18]. Sonic flow affects the ability of diffusion and penetration in that area, causing cells to be relatively active in achieving the desired treatment effect. In addition to providing thermal energy, applying the cavitation effect means that ultrasound can create tiny air bubbles in the body [19]. The air bubbles can increase the sonic flow mentioned above [20]. For this reason, making lipid-based nanomaterials with gaseous or hollow structures can alter ultrasonic contrast; therefore, developers use them [21]. Improvements can be made to the ultrasound detection method through nanomaterials. Intriguingly, nanomaterials have also been demonstrated to be helpful for drug-carrying therapeutic applications [22]. As a result, ultrasound can also provide precision treatment and be used as a detection method. The most exciting aspect of this method is that it can enhance the effectiveness of brain detection and drug delivery by effectively breaking the blood–brain barrier. The synergistic effect between nanomaterials and ultrasound therapy is the focus of this review. We mainly cited ultrasound-related topics from 2018 to 2022 and selected articles around drug screening, cellular mechanisms, and therapeutic processes. Those references were compiled through the NCBI PubMed and WOS (Web of Science) databases. In ultrasound-based diagnosis and drug delivery, microbubbles, nanobubbles, and liposomes are the common contrast reagents. By using these novel nanomaterials, modifications to ultrasonic applications can be achieved. This paper primarily discusses the contrast reagents currently applied to ultrasound to provide a complete update on image presentation and treatment [23].

## 2. Ultrasound and Nanomedicine Screening

For the purposes of diagnosing injuries to internal tissues, ultrasound is helpful in the diagnosis and treatment of sports injuries. A combination of auscultation and X-ray can be used to diagnose pericardial effusion, replacing invasive diagnostic methods. Since the resolution of ultrasound in image presentation has been the biggest challenge, studies have shown that ultrasound has only been able to diagnose panic tumors since about the 1970s [24]. The research conducted by R. Langer suggests that ultrasound can affect the rate of drug release and can enhance the time required for drug penetration through the skin. With the development of drugs and nanoscale carriers, ultrasound technology can be further used for diagnostic and therapeutic purposes. The metabolism of drug carriers by ultrasound suggests the potential for contrast reagents to be used as topical drugs to reduce side effects. Herein, we list references to ultrasonic techniques for cancer-targeted drug delivery systems published over five years (Table 1).

### 2.1. Ultrasonic Technique

**Figure 1:**
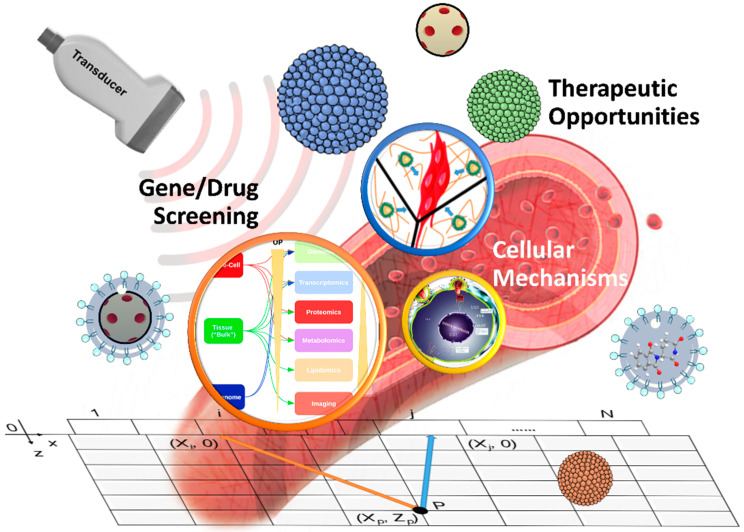
Schematic overview of high-intensity focused ultrasound for tumor treatment. Its primary treatment items are gene/drug screening, cellular mechanism, and therapeutic progress.

The sound waves audible to the human ear are approximately 20 to 20,000 Hz (hertz). In other words, sound waves with more than 20,000 Hz frequency are “ultrasonic waves” and can be transmitted through vibrations in various media [25]. By utilizing a transducer or the probe of a piezoelectric crystal, ultrasound can be converted into electrical energy. The ultrasonic wave is conducted at the interface of different medium densities, and the probe reflects the energy [26]. The strength of this reflection is proportional to the electric signal generated [27]. The resulting electric signal transformation is presented as a grayscale image for subsequent visual interpretation [28]. Presently, ultrasound is considered a noninvasive, radiation-free form of detection that can be used to diagnose and treat diseases and disabilities and promote rehabilitation (Figure 1). Water-containing tissues in human tissues are the most effective conductors of sound waves, other than the lungs, which contain bones and gas [29]. When lung diseases occur, the related tissue fluid can serve as a valuable detectable material even though the lungs are not the best conducting medium for ultrasound.

### 2.2. Ultrasonic Drug Screening

The stability and permeability of a drug determine its effectiveness. During cancer treatment, patients usually experience intense side effects from their medications. The interstitial pressure generated by the tumor at a particular location may also limit the drug’s effectiveness, permitting some cancer cells to survive, which may lead to drug-resistant cells and side effects in normal cells. Therefore, “encapsulation and selective drug delivery” is a strategy for effectively solving these problems, and ultrasound and nanobubbles could be used to accomplish this. It has been proposed that liposomes and micelles may sequester hydrophobic compounds within their lipophilic membranes (liposomes) or cores (micelles), while liposomes might sequester hydrophilic compounds within their aqueous interiors. These vesicles prevent the general or premature release of the drug. Using cavitation and thermal methods, Paul L. Carsona’s research team demonstrated that microbubble distribution could be controlled in terms of the spatial distribution of bubbles, and bubble vaporization (droplet formation) could be accomplished through acoustic droplet vaporization [30]. Such bubbles can remain in mouse models for several days. Of note, nanobubbles can provide acoustic imaging via ultrasound and may thus serve as therapeutic and diagnostic tools. By combining liposomes with microvesicles, in which gas and stabilization oil are introduced into standard liposomes, such “lipid globules” can be seen as a blend of pure liposomes and microvesicles and can also be used as ultrasound contrast agents. Furthermore, when the microbubbles carry the lipid spheres along with the drug, they are doubly effective drug delivery vehicles, serving as vehicles and activators for ultrasonic drug delivery. Some medicines can spontaneously attach to liposomes and microvesicles. A good example is the binding of negatively charged DNA to liposomes and microvesicles with cationic surfactants. By cavitating, the drug can be released from liposomes or microbubbles. In this way, combining the packaging method of bubbles and ultrasound could be suitable as a screening platform.

**Table 1:** Ultrasonic techniques for cancer-targeted drug delivery system.

**Even**	Model	Cancer Types	Delivery Vehicles	Ultrasonic Frequency	Effect	Ref.
Biopsy	Patients	Breast cancer	Microbubbles	4.5 to 15 MHz	Enhanced preoperative axillary staging	[31]
In vitro/in vivo	AsPC1/transgenic pancreatic cancer mouse	Pancreatic ductal adenocarcinoma	Microbubbles	21 MHz	Increased Thy1 expression in PDAC	[32]
In vivo	MDA-MB-231, MCF-7, MCF-12A	Breast cancer	Microbubbles	5 to 7.5 MHz	Enhanced drug response	[33]
In vitro/in vivo	PC-3	Prostate cancer	Microbubbles	1 MHz	Enhanced Efficacy of Photodynamic Therapy	[34]
In vitro/in vivo	Bel-7402 Bel-7402, SKOV-3, MB-231	Cervical, ovarian, and breast cancer	Microbubbles	0.8 to 3.5 MHz	Enhanced and synergistic chemotherapy	[35]
In vitro/in vivo	MCF-7	Breast cancer	Microbubbles	2 to 10 MHz	Enhancing therapeutic efficacy	[36]
In vitro/in vivo	HT-29	Colorectal cancer	Microbubbles	1 to 12 MHz	Overcomes Multidrug Resistance	[37]
In vivo	PC-3, LNCaP	Prostate cancer	Microbubbles	5 to 10 MHz	Enhances the detection of tumor cells	[38]
In vitro/in vivo	Walker-256 BC	Breast cancer	Microbubbles	1.5 to 7.5 MHz	Inhibiting the tumor growth	[39]
In vitro	LS174T, CT26	Colon cancer	Microbubbles	3.2 MHz	Enhances the monitoring of the therapy	[40]
In vitro	MDA-MB-231	Breast cancer	Microbubbles	9 MHz	Optimization of the target condition	[41]
In vitro	HUVECs	Endothelial cells	Microbubbles	0.4 to 8.5 MHz	Enhancing the efficiency of labeling	[42]
In vitro/in vivo	CT26	Colon cancer	Microbubbles	6.5 MHz	Induce photothermal therapy activity	[43]
In vitro	PC-3, LNCaP	Prostate cancer	Microbubbles	5 to 12 MHz	Enhancing the efficiency of labeling	[44]
In vitro/in vivo	MDA-MB-231	Triple-negative breast cancer	Microbubbles	1.5 to 12.5 MHz	Enhancing the efficiency of labeling	[45]
In vivo	MDA-MB-231	Breast cancer	Microbubbles	32 MHz	Enhancing the efficiency of radiation therapy	[46]
In vivo	VX2	Liver cancer	Microbubbles	3 to 9 MHz	Improved the antitumor effect	[47]
In vitro/in vivo	OVCAR-3, 4T1	Breast cancer, ovarian cancer	Microbubbles	6 to 10 MHz	Enhancing the delivery of drugs	[48]
In vitro	MOLM-13	Leukemia	Microbubbles	1.108 MHz	Enhanced the therapeutic effectiveness of treatment	[49]
In vitro/in vivo	Bel-7402	Liver cancer	Microbubbles	1 MHz	Improved diagnostic accuracy and synergistic treatment	[50]
In vitro/in vivo	TRAMP	Prostate cancer	Microbubbles	7 MHz	Improved the efficiency of diagnosis	[51]
In vivo	VX2	Liver cancer	Microbubbles	1 MHz	Enhanced the response to treatment	[52]
In vivo	MDA-MB-231	Breast cancer	Microbubbles	8 MHz	Enhanced the efficacy of therapy	[53]
In vitro/in vivo	SVR	Cholangiocarcinoma	Microbubbles	40 MHz	Enhancing diagnostic and therapeutic capabilities	[54]
In vitro/in vivo	KHT-C	Fibrosarcoma	Microbubbles	4 to 5.2 MHz	Improved the efficiency of diagnosis	[55]
In vitro/in vivo	MDA-MB-231	Breast cancer	Microbubbles	7 MHz	Improved the efficiency of diagnosis	[56]
In vivo	Spontaneous tumor mice	Liver cancer	Microbubbles	1.6 MHz	Improved the efficiency of diagnosis	[57]
In vitro/in vivo	MC38	Colon cancer	Microbubbles	4 MHz	Enhanced immune response	[58]
In vitro	PaCa-2	Pancreatic cancer	Microbubbles	2 MHz	Enhanced the efficacy of therapy	[59]
In vivo	MDA-MB-231	Breast cancer	Microbubbles	20 MHz	Improve the efficiency of diagnosis	[60]
In vitro/in vivo	VX2	Liver cancer	Microbubbles	3.5 MHz	Enhanced drug delivery and therapeutic effect	[61]
In vivo	Tumorigenesis induced by diethylnitrosamine	Liver cancer	Microbubbles	21 MHz	Enhanced the therapeutic effect	[62]
In vivo	RT112	Bladder cancer	Microbubbles	8 MHz	Enhanced the therapeutic effect	[63]
In vitro/in vivo	U14	Cervical carcinoma	Microbubbles	18 MHz	Enhanced the therapeutic effect	[64]
In vivo	VX2	Liver cancer	Microbubbles	9 MHz	Improved the efficiency of diagnosis	[65]
In vivo	PC-3	Prostate cancer	Microbubbles	25 MHz	Enhanced the therapeutic effect	[66]
In vivo	PANC-1	Pancreatic cancer	Microbubbles	4 MHz	Enhanced the therapeutic effect	[67]
Biopsy	Patients	Breast cancer	Microbubbles	6 to 15 MHz	Improved the efficiency of diagnosis	[68]
In vitro/in vivo	SCC-7	Mouse squamous cell carcinoma	Microbubbles	1 MHz	Enhanced the therapeutic effect	[69]
In vivo	MDA-MB-231	Breast cancer	Microbubbles	21 MHz	Improved the efficiency of diagnosis	[70]
In vitro/in vivo	MDA-MB-231	Breast cancer	Microbubbles	25 MHz	Enhanced the therapeutic effectiveness of treatment	[71]
In vivo	PC-3	Prostate cancer	Micro/nanobubbles	18 MHz	Improved the efficiency of diagnosis	[72]
In vitro/in vivo	C6	Glioma	Micro/nanobubbles	1 to 10 MHz	Antitumor activity	[73]
In vitro/in vivo	MDA-MB-468	Breast cancer	Microbubbles/liposomes	1 MHz	Improved the delivery of materials	[74]
In vitro/in vivo	Cal-27, OECM-1	Oral cancer	Nanobubbles	7 MHz	Promoted the release of reactive oxygen species (ROS)	[75]
In vitro	CT26	Colon cancer	Nanobubbles	13 to 24 MHz	Enhanced the therapeutic effect	[76]
In vitro/in vivo	SKBR3	Breast cancer	Nanobubbles	22 MHz	Enhanced the targeting precision	[77]
In vitro/in vivo	MDA-MB-231	Breast cancer	Nanobubbles	3 to 9 MHz	Enhanced the precision and accuracy of targeting and diagnosis	[78]
In vitro/in vivo	4T1	Breast cancer	Nanobubbles	1 MHz	Enhanced drug delivery and therapeutic effect	[79]
In vitro/in vivo	U87, MDA-MB-231	Glioblastoma, breast cancer	Nanobubbles	7.5 MHz	Improved diagnostic accuracy and synergistic treatment	[80]
In vitro/in vivo	OVCAR-3, 4T1	Breast cancer, ovarian cancer	Nanobubbles	12 MHz	Enhancing the delivery of drugs	[48]
In vitro/in vivo	MCF-7, MDA-MB-468	Breast cancer	Nanobubbles	18 to 21 MHz	Enhancing diagnostic and therapeutic capabilities	[81]
In vitro/in vivo	PC-3	Prostate cancer	Nanobubbles	12 MHz	Enhancing the sensitivity of diagnosis	[82]
In vitro/in vivo	LNCaP, C4-2, and PC-3	Prostate cancer	Nanobubbles	13 to 24 MHz	Improved the efficiency of diagnosis	[83]
In vitro/in vivo	MDA-MB-231, MDA-MB-468	Breast cancer	Nanobubbles	13 to 24 MHz	Enhanced drug delivery and therapeutic effect	[84]
In vivo	PC-3	Prostate cancer	Nanobubbles	18 MHz	Improved diagnostic accuracy and synergistic treatment	[85]
In vitro/in vivo	4T1	Breast cancer	Nanobubbles	7.5 MHz	Enhanced drug delivery and therapeutic effect	[86]
In vivo	LN-229	Glioblastoma	Nanobubbles	12 MHz	Improved the efficiency of diagnosis	[87]
In vitro/in vivo	MDA-MB-231	Breast cancer	Nanobubbles	18 to 38 MHz	Enhanced drug delivery and diagnosis	[88]
In vitro/in vivo	Mia-Paca2	Pancreatic cancer	Nanobubbles	7.5 MHz	Improved diagnostic accuracy and synergistic treatment	[89]
In vitro/in vivo	4T1	Breast cancer	Nanobubbles	7.5 MHz	Improved the efficiency of diagnosis	[90]
In vitro	MiaPaCa-2, Panc-1, MDA-MB-231, AW-8507	Pancreatic cancer, breast cancer, head, and neck cancer	Nanobubbles/liposomes	1 MHz	Improved the efficiency of diagnosis	[91]
In vitro/in vivo	MDA-MB-231, B16F10	Breast cancer, melanoma	Liposomes	1 to 12 MHz	Improved diagnostic accuracy and synergistic treatment	[92]
In vitro	SKOV3, A549	Ovarian cancer, lung cancer	Liposomes	5 to 12 MHz	Enhanced drug delivery and therapeutic effect	[93]
In vitro/in vivo	MDA-MB-231	Breast cancer	Liposomes	1.3 MHz	Enhanced drug delivery and diagnosis	[94]
In vitro	NCI-N87	Gastric cancer	Liposomes	10 MHz	Enhanced drug delivery and diagnosis	[95]
In vivo	4T1	Breast cancer	Liposomes	40 MHz	Improved diagnostic accuracy and synergistic treatment	[96]
In vivo	GL261	Glioma	Liposomes	1 to 2 MHz	Enhanced drug delivery and therapeutic effect	[97]

## 3. Cellular Mechanisms

Ultrasound-induced bubble cavitation is typically induced by the oscillating motion of an acoustic fluid, allowing efficient diffusion of molecules. It is thought that there are two ways to generate convection, microflow and sound pressure, to enhance the liquid’s convection and increase the drug transport rate. As an additional benefit, ultrasound can cause drug release through cavitation of drug carriers caused by vesicle disruption. With ultrasound being used in conjunction with bubbles, it has now been found to be clinically effective for treating brain disorders and drug resistance.

### 3.1. Blood–Brain Barrier Opening

The blood–brain barrier (BBB) is tissue containing capillaries and endothelial cells within the brain surrounded by astrocytes, forming a natural barrier between the plasma and the cerebrospinal fluid of the central nervous system. Typically, the BBB prevents brain infection by bacteria or viruses and is selective for macromolecules or water-soluble molecules, generally permeable only to nutrients and fat-soluble molecules less than 400 Da [5]. Consequently, effective treatments for brain-related diseases are often lacking. In addition to glioblastoma, other types of cancer, such as lung cancer, breast cancer, and melanoma, can metastasize to the brain. Bubbles and ultrasound allow the BBB to be briefly opened [98]. This discovery may provide new insight into treating diseases associated with the brain.

#### 3.1.1. Microbubbles

**Figure 2:**
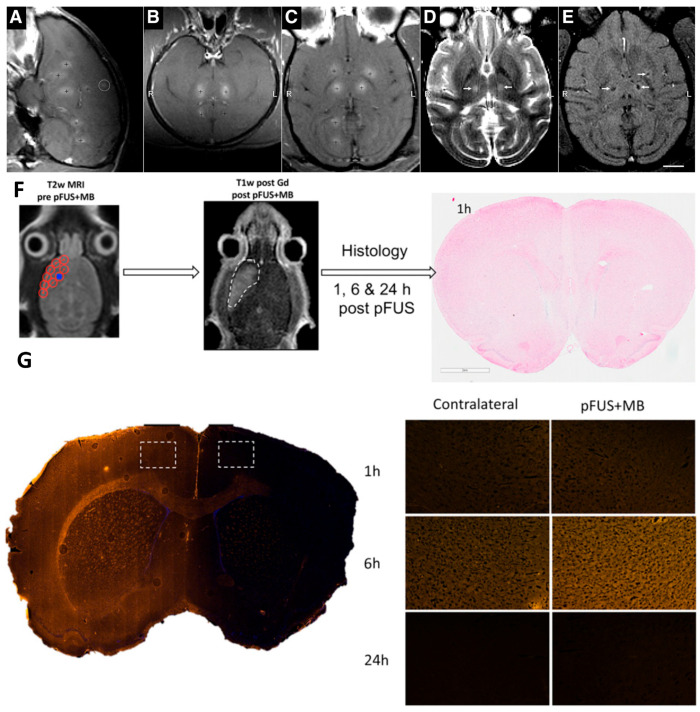
Synergistic effect of focused ultrasound and MRI for BBB theranostics. (**A**–**C**) BBB disruption in monkey brain tissue following focused ultrasound and microbubble targeting of a single tissue site (white circle; R, right and L, left). (**D**,**E**) After the interruption of the BBB presentation, an MR contrast agent was added, and this imaging agent penetrated the brain (white arrows). (**F**) Experimental design for the histological analysis of a rat brain treated with focused sonication. Targeted concentrated ultrasound on spots approximately 2 mm in diameter (red circles) and a Gd-enhanced T1-weighted image after using focused ultrasound. The white dashed line outlines the contrast-enhanced results, and no apparent damage was observed on H&E. (**G**) After the focused ultrasound and microbubble treatment, the white dashed rectangles indicate the accumulation of albumin occurring through the open exudative BBB. Adapted with permission from Refs. [99,100]. Copyright 2012 American Association for Cancer Research and 2016 Proceedings of the National Academy of Sciences of the United States of America.

Microbubbles are generally defined as measuring between 0.5 and 10 μm in diameter. Microbubbles are effective reflectors of ultrasound energy and can serve as contrast agents in imaging due to their acoustic impedance and compressive strength. Microbubbles can also be used for the ultrasonic treatment of cavitation nuclei and lower the threshold of ultrasonic cavitation. Researchers have demonstrated, for the first time, that microbubbles can be used to open the local blood–brain barrier without causing any tissue damage based on low-intensity focused ultrasound experiments. Due to the ability of bubbles to carry drugs, several studies have focused on optimizing the treatment of brain diseases [99]. The therapeutic diagnosis of BBB using focused ultrasound and MRI can improve imaging diagnosis within the target area (Figure 2A–E). After adding microbubble material, treating the brain tissue with focused ultrasound, and performing minor image enhancement in the brain groove, the BBB structure overlapping the lateral ventricle is loosened near the most superficial position [100]. However, even if the same exposure level is used for each sonication, the magnitude and extent of the additional damage will vary among different tissues; in this way, the resolution of the ultrasound diagnosis can be enhanced (Figure 2F). Furthermore, a T2-weighted image analysis with MRI showed that, although edema developed at two targets in the thalamus, it became relatively apparent in the putamen upon T2-weighted imaging. There was no obvious brain damage after H&E staining, indicating that using microbubbles can improve the efficiency of ultrasound and provide specific diagnosis and treatment for the BBB (Figure 2G).

#### 3.1.2. Nanobubbles

**Figure 3:**
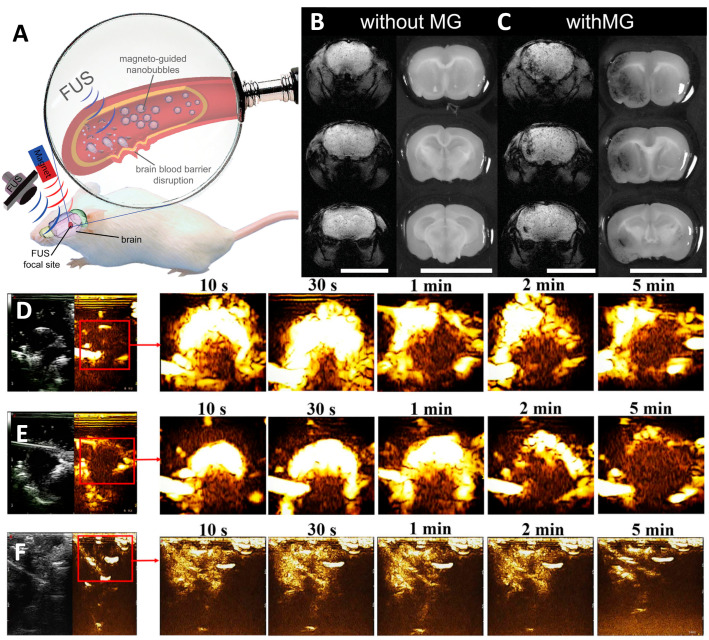
(**A**) Nanobubbles can be used to diagnose locally disrupted BBB and accumulate due to magnetic guidance (MG). (**B**) Representative images of T2 gradient brain slices and their corresponding dye-treated brain tissue slices, used to assess the efficiency of bubble-free and magnetically guided brain tissue BBB disruption. (**C**) Biosafety induced by focused ultrasound compared to different degrees of BBB destruction caused by nanobubbles. Contrast-enhanced ultrasound imaging of brain tumors at other timepoints (10 s to 5 min) before and after injection of (**D**) nanobubbles and (**E**) commercial SonoVue. (**F**) Brain cavity ultrasound images at the same timepoint before and after the injection of NBs. Red squares show image enhancement of specific tissue sites. Adapted with permission from Refs. [103,104]. Copyright 2020 Elsevier and 2014 John Wiley and Sons.

Nanobubbles are approximately 200–400 nm in size compared to microbubbles. There is evidence that nanobubbles contribute to BBB opening [101]. Despite having a similar structure to ice or gas hydrates, they are no easier to burst than microbubbles [102]. Moreover, nanobubbles can be delivered more easily into higher-density tissues than microbubbles [103]. Therefore, their therapeutic effects on the brain have been extensively explored with magnetic guidance (Figure 3A). An in vivo T2-weighted MRI was used to observe and assess nanobubbles and MRI-guided brain tissue distribution (Figure 3B,C). No significant changes were observed in the histology of nanobubble brains without magnetic guidance (MG). In contrast, a T2-MRI of two brain tissue sections confirmed that the BBB disrupted penetration and nanobubble deposition when applied using MG [104]. However, the ultrasound signals also confirmed the high correlation between the nanobubbles and the distribution of ultrasound images, significantly improving the ultrasound contrast of the brain images (Figure 3D–F).

### 3.2. Ultrasound-Induced Cellular Mechanism

#### 3.2.1. Drug Resistance

**Figure 4:**
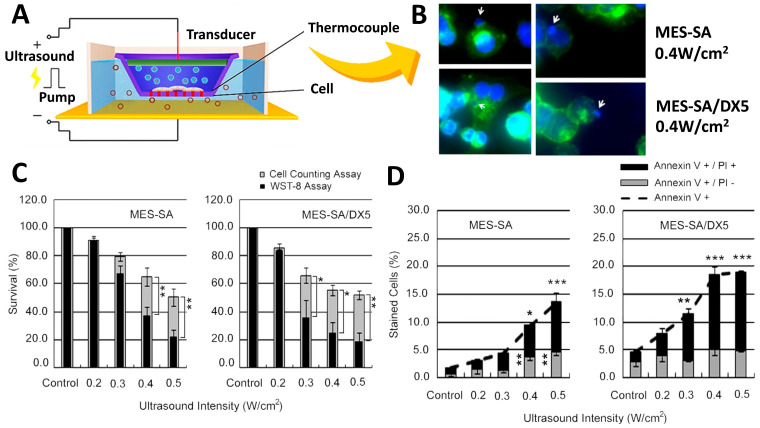
(**A**) Schematic of the apparatus used to expose cells to ultrasound in vitro. Cells are cultured in transwells, and liposomes deliver siRNA or shRNA to inhibit the drug resistance of cancer cells. (**B**) After sonication at 0.4 W/cm^2^ for 24 h, the differences and sensitivity results of drug-sensitive uterine sarcoma cell lines before and after adding lipospheres (DX5) were observed with conjugate focus microscopy. White arrows indicate nuclear budding and demonstrate the inhibition of cells after adding lipospheres. Cell viability was assessed by: (**C**) WST-8 and cell-counting assays, showing that, after adding DX5 lipospheres, the viability of the cells was significantly inhibited. (**D**) Flow cytometric analysis of FITC-labeled Annexin V showing the cytostatic conditions. Asterisks (*) indicate the statistical significance of the difference between the absolute percentages obtained from cell counting assays, * *p* < 0.05, ** *p* < 0.1, and *** *p* < 0.01 considered significant. Adapted with permission from Ref. [107]. Copyright 2012 PLOS.

Clinical drugs are not able to effectively cure cancer due to multidrug resistance. Tumor-mediated tumor microenvironments contain tumor-associated stromal cells, which are capable of facilitating drug resistance in addition to the original mutation of somatic cells, which is the primary cause of drug resistance. Due to the permeability and stability of the drug itself, and the spatial complexity of blood vessels and tissues, etc., the drug cannot reach the critical site at an appropriate dose to eliminate cancer cells, resulting in residual disease. As a result, some cancer cells become drug-resistant. Researchers have found that the p-glycoprotein transporter is overexpressed in cancer cells, and this causes drugs to be transported out of the cells, resulting in drug resistance. An shRNA that encapsulates p-glycoprotein shRNAs and preserves the relative concentration of DOX, which can improve the effectiveness of DOX therapy for patients with breast cancer. Furthermore, anti-cell-death-related genes, such as BCL-2, appear to be key molecules in the enablement of cancer cells to resist drug-induced death. Encapsulating siRNA for BCL-2 in bubbles has been shown to reduce resistance to paclitaxel (PTX) in hepatocellular carcinomas. PTX resistance has also been reduced by targeting si-survivin RNA in lung cancer [105]. It has been demonstrated that antibody labeling inhibits drug resistance by encapsulating siRNA or shRNA and effectively delivers bubbles to tumor cells. In epithelial ovarian cancers, PTX bubbles containing the MUC16 antibody can effectively inhibit resistance to PTX [106]. In this regard, wrapping bubbles and targeting markers differently could address the dilemma associated with drug resistance (Figure 4A). Upon exposure from drug-sensitive uterine sarcoma cell line cells (MES-SA) with differential sensitivities, after adding lipospheres to the US, it was found that cell viability 24 h after sonication, and cell counts for both cells, decreased in an intensity-dependent manner, according to WST-8 assay cells (Figure 4C) and Annexin V (Figure 4D). Moreover, the double staining of adherent cells with Hoechst 33342 and Alexa Fluor 488-conjugated wheat germ agglutinin revealed the appearance of nuclear buds, which were seen during translocation to cell division (Figure 4B). Based on these results, the inhibition of cell division was observed in sonicated MES-SA cells due to liposphere treatment [107].

#### 3.2.2. Physiochemical Mechanism

Ultrasound is a high-frequency sound wave generated by passing an electric current through a crystal. This high-speed vibration causes vibration and heat generation when it meets fibrous tissue. Because its therapeutic effect can penetrate subcutaneously, it can be used for deep heat therapy. The ultrasound used in rehabilitation is the same as that used in obstetrics and gynecology prenatal examinations or other medical examinations. The frequency is different, so the effect is different. Ultrasound can relax the adherent tissue, increase the tissue’s extensibility, improve the tissue’s elasticity, promote local blood circulation, effectively reduce inflammation, provide pain relief, etc. Ultrasound has both thermal effects and nonthermal effects. When ultrasound enters the human body, the thermal effect is converted into heat energy, resulting in blood circulation, increased soft tissue ductility, pain relief, muscle spasm, etc. It is the deepest heat penetration possible among all thermal therapies. The nonthermal effects are mainly mechanical shock wave effects, including cavitation, acoustic streaming, and micro streaming.

**Figure 5:**
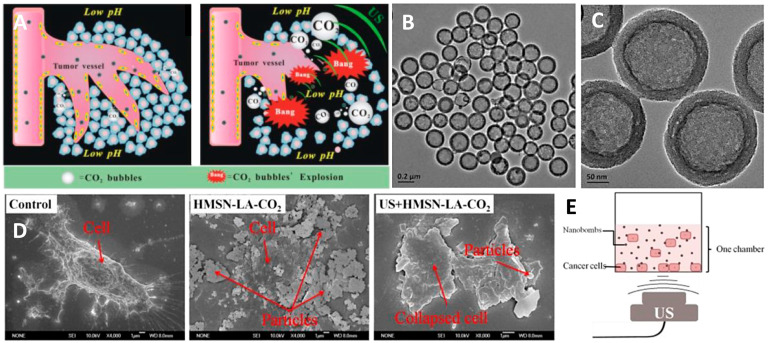
(**A**) Schematic diagram of CO_2_ nanobubble physiotherapy mechanism. The nanobomb system can be driven by ultrasound to generate thermal energy through the cavitation effect. (**B**,**C**) Characterization of nanobombs based on carbon dioxide nanobubbles in TEM images. (**D**) Therapeutic outcomes were assessed in vitro by inducing apoptosis in PANC-1 pancreatic cancer cells using a constructed CO_2_ bubbling-based system. (**E**) Schematic diagram of the measuring apparatus for in vitro PANC-1 cell experiments. Adapted with permission from Ref. [108]. Copyright 2015 Ivyspring International Publisher.

Heat therapy is a practical tool for tumor treatment, but its mechanism of action has long been poorly understood. In recent years, with the re-emergence of heat therapy as one of the hotspots of research, significant progress has been made in studying the mechanism of action for heat therapy. Studies have shown that subthermal thermotherapy mainly kills cells by inducing apoptosis, whereas hyperthermia directly causes cell necrosis. Heat-induced apoptosis is achieved through the mitochondrial and/or death receptor pathway, and increases in oxidative stress and intracellular Ca^2+^ play an essential role in inducing apoptosis. Some combination therapies based on the mechanism of heat-induced apoptosis, such as heat therapy combined with gene therapy, oxidative stress, Ca^2+^-targeted therapy, and the reduction of extracellular pH, can significantly enhance the effect of heat therapy. Heat shock proteins (HSPs) are a class of functionally related proteins whose expression increases when cells are exposed to elevated temperatures or other stresses. These HSPs, which are widely found in eukaryotic and prokaryotic cells, are thought to help stabilize the structure of proteins to maintain protein activity when exposed to environmental stresses that tend to cause protein inactivity. Like other macromolecular HSPs, small heat shock protein-Hsp27 also functions as a molecular chaperone, preventing the irreversible aggregation of unfolded proteins. Hsp27 is abundantly expressed in many tumor cells, increasing cellular resistance to heat and oxidative stress and inhibiting planned cell death (apoptosis). The physiotherapy technology route can utilize a pH/temperature-responsive delivery system and mechanically disrupt the tumor and surrounding blood vessels of tumor cells via intravascular mechanisms (Figure 5A) [108]. Heat can be generated to treat tumors through the bubble cavitation effect triggered by ultrasound irradiation, and CO_2_ bubbles induced in the lower pH microenvironment within the tumor will mostly dissolve in the blood or interstitial fluid compared to without ultrasound. This leads to the prominent inhibition of inertial cavitation (nanobombs) and a thermal effect to induce apoptosis (Figure 5E). The CO_2_ bubbles were characterized with TEM images (Figure 5B,C) and transported to a cancer cell to observe the collapsed cells (Figure 5D).

#### 3.2.3. Biological Mechanism

It has been shown that ultrasound might induce cell death at low intensities, even when the temperature is not significantly elevated. This subsection identifies and characterizes some factors that enhance these effects, as well as those that inhibit those factors. Several methods were applied to study the biological effect mechanisms and explore the biological effects.

**Figure 6:**
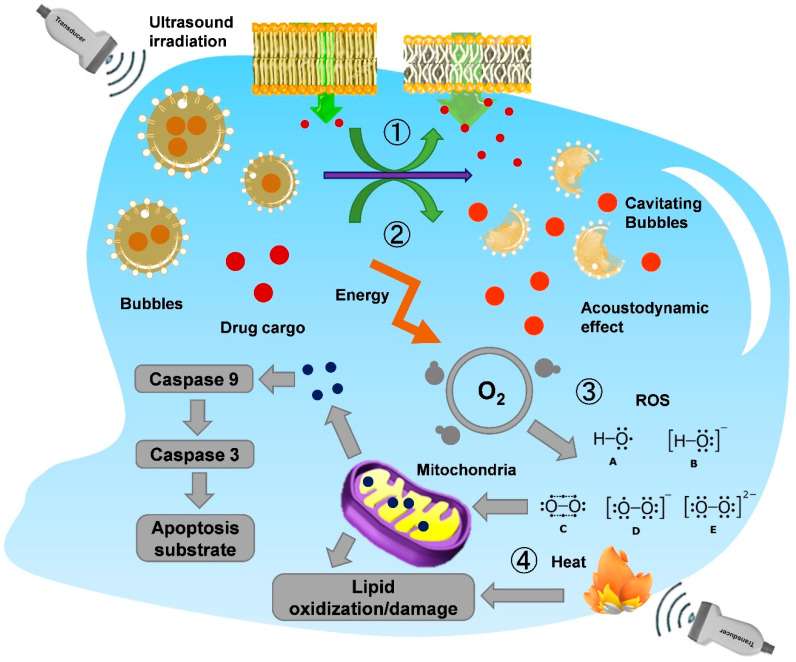
Schematic diagram of the various cellular change mechanisms induced by ultrasound. ① Cavitation caused by ultrasonic waves induces the rupture of bubble contrast agents in cells. ② Drug cargo is released, and acoustically powered drug delivery is provided. ③ Intracellular reactive oxygen species (ROS) may also be involved in cytotoxicity due to ultrasound excitation and generation. ROS-mediated mitochondrial membrane damage and the release of cytochrome c induce apoptosis. ④ There is also membrane damage due to the treatment with high-frequency sound waves since the unavoidable heat generation.

The possible cellular mechanisms of synergy between ultrasound energy and certain drugs, especially anticancer drugs, identified in a biological mechanism setting are: (1) ultrasound increasing permeability, characterized by increased cellular uptake of the drug; (2) ultrasound increasing cellular sensitivity to the drug, an enhancement also known as the acoustodynamic effect; (3) high-frequency ultrasound producing irreversible partial damage; and (4) the thermal effect. However, it has been suggested that these mechanisms largely overlap, and that their proportions depend on many factors, including the type (Figure 6). It is well known that cells expand due to a loss of tension, and that the cell membrane is a significant participant in this event. The energy of ultrasound causes the cell membrane to become relaxed, thereby increasing the penetration of the drug into the cell. This physical change to the cells, provided by ultrasound energy, is also known as the acoustodynamic effect. In addition to changes in drug permeability, current studies have demonstrated that the membrane damage and repair mechanisms of cells are affected in the ultrasound environment. Prolonged exposure to ultrasound causes a decrease in cellular tension and increases membrane damage, which decreases the membrane repair capacity and causes cell death.

This finding may help elucidate the mechanical nature of ultrasound-induced biological effects and the cellular response to these effects. These effects may also be clinically beneficial when coupling ultrasound therapy with hypoosmotic fluid delivery to the target tissue, especially in cancer therapy. In ultrasound therapy, which has been used as a heating device for thermal therapy in cancer treatment, recent studies have shown that nonthermal low-intensity ultrasound can produce apoptosis in vitro and in vivo under certain conditions. However, some of the significant problems are their low yield compared to other modalities and the predominance of cell lysis rather than apoptosis as a form of cell death in most cases.

## 4. Ultrasonic Diagnosis

Ultrasonic diagnosis has gradually evolved into a mature technology used for disease monitoring and diagnosis in the medical field. Due to the lack of resolution in ultrasound development, contrast agents were developed to enhance the recognition of ultrasound images. Ultrasonic contrast agents are micro/nano-sized bubbles smaller than red blood cells. When the bubble shell dissolves, the gas inside will not react with the human body but instead will be discharged through breathing via pulmonary circulation. The liver and kidney metabolize the lipid-based molecules, which is relatively safe. Another advantage is that one can see the boundary and scope of a lesion under ultrasound and have a clear target during treatment, which can increase the accuracy of the radiofrequency ablation of liver cancer. Today, this technology has been applied to the treatment of liver tumors, called radiofrequency (or microwave) therapy, under the guidance of ultrasound imaging enhancement, which can significantly improve the integrity and success rate of the treatment. Contrast agents have been used for many years and are essential for monitoring small liver tumors or liver cancer surgical guidance. Its accuracy is similar to CT and MRI; it is not limited to the liver and is also applicable to most celiac diseases. High-risk groups should be screened regularly to determine the best time for treatment.

### 4.1. Liposomes

**Figure 7:**
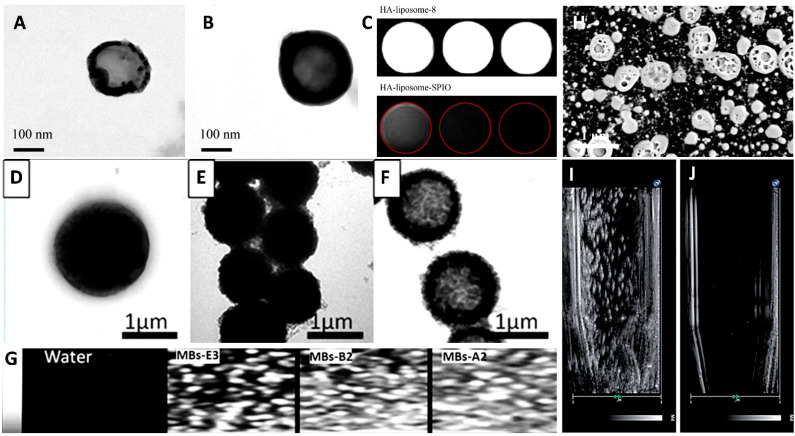
Different types of lipid spheres are used as contrast agents for ultrasound imaging. (**A**) Hydroxyapatite-coated liposomes and (**B**) hydroxyapatite-coated liposomes with superparamagnetic iron oxide. (**C**) MRI analysis with different liposomes. Microbubbles were modified with (**D**) polyamine salt, (**E**) magnetic polyamine salt, and (**F**) Fe_3_O_4_ nanoparticles. (**G**) Ultrasonic contrast images with microbubbles. (**H**) SEM images of nanobubbles. The ultrasonic wave was treated to a break of nanobubbles for (**I**) 0 s to (**J**) 5 min. Adapted with permission from Refs. [111,112]. Copyright 2011 and 2016 Elsevier and 2018 The Royal Society of Chemistry.

Liposomes consist of bilayers of lipids that contain phospholipids and cholesterol and are made up of hydrophilic nucleospheres measuring approximately 50–200 nanometers in size (Figure 7A,B). Due to their biocompatibility and enhanced targeting abilities, nanoparticles, such as daunorubicin and polyethylene glycol, are commonly used in cancer treatment to encapsulate and deliver drugs in vivo [109]. Moreover, liposomes can be used as contrast agents to assess differences between the distributions of drugs across tissues. Studies have shown that liposomes modified with cRGD peptide can also form a visual monitoring function with neutrophils with acoustic parts, subsequently accumulating in the tumor area to enhance ultrasound stimulation on the tumor [110]. Although the liposome itself cannot provide an ultrasound imaging diagnosis, by combining it with magnetic materials, the liposome can be analyzed with MRI technology (Figure 7C) [111].

### 4.2. Microbubbles

Microbubbles are shown above to illustrate their role in the packaging of drugs. Several studies have shown that, when microbubbles are in vivo, they will reflect ultrasonic sound waves by changing their shape (Figure 7D–F). Consequently, these changes can form distinct contrast points in high-sonic images that can be used for diagnostic purposes to identify lesions. For enhanced ultrasound diagnostics, products using microbubbles are approved by the Food and Drug Administration (FDA), including Optison™, Lumason^®^, and Definity^®^. Microbubbles with a diameter of 2 to 8 µm are injected intravenously into the patient and then receive signals through an ultrasonic probe. The dynamic image features of the liver tumor’s blood vessels will be displayed on the ultrasonic screen [112]. Due to the improved toughness of microbubbles, microbubbles can oscillate at lower energy, and echoes can be repeatedly generated to help observe images. The retention time in the liver can be as long as 1 h. (Figure 7G)

### 4.3. Nanobubbles

Nanobubbles, like microbubbles, can act as contrast agents when exposed to ultrasound waves (Figure 7H). Due to the small size of microbubbles, this makes it easier for them to penetrate and remain in the tumor vascular area in vivo, which leads to better image recognition results in ultrasound imaging. The prostate-specific membrane antigen (PSMA) is the most commonly used marker for diagnosing prostate cancer. PSMA-nanobubbles are more sensitive in imaging than microbubbles formed with Lumason^®^ [82,113]. A similar approach has been used to diagnose various cancers using nanobubbles as targeting tools (Figure 7I,J). Using the CA-125 antibody as a marker can enhance the accumulation and signaling of nanobubbles in epithelial ovarian cancer. Labeling with ErbB2 or HER2 antibodies can improve the sensitivity and specificity of breast cancer detection. It was recently found that using Cyanine 5.5 as a marker for nanobubbles could also be used to further improve the ability to image PSMA antibody labeling, providing it the function of dual ultrasound and fluorescence imaging.

## 5. Ultrasonic Therapy

The utility of ultrasound and contrast reagents in the delivery and release of drugs in cancer treatment has already been proven. This section presents several examples associated with the delivery and release of common clinical drugs. Combined with these FDA-approved drugs, ultrasound contrast agents have diagnostic and therapeutic functions. A recent five-year study of these FDA-approved chemotherapeutic agents is listed in Table 2 to illustrate the importance of combining the drug with the contrast reagent.

### 5.1. FDA-Approved Drugs

#### 5.1.1. Paclitaxel

**Figure 8:**
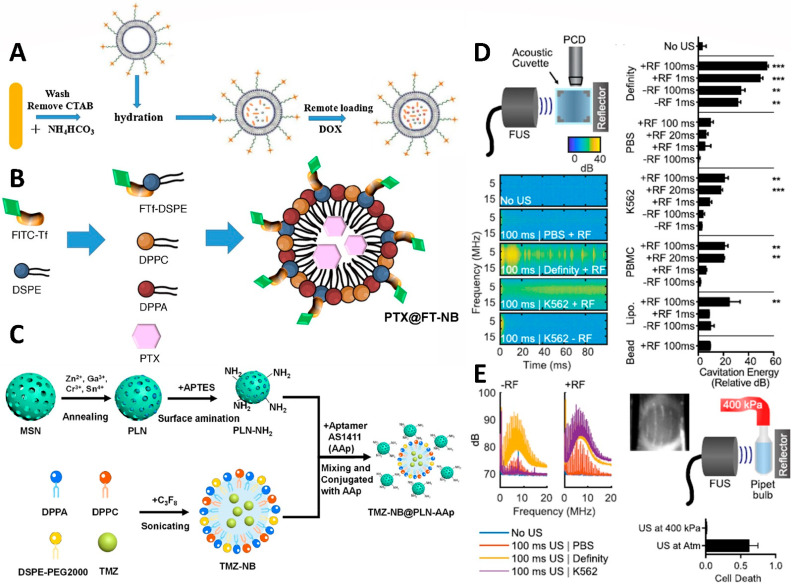
Schematic illustrations showing the structure and biological functions of the (**A**) bubble-generating liposomes loaded with doxorubicin, (**B**) nanobubble-embed PTX, and (**C**) inorganic/nanobubble-conjugated nanocomposites with temozolomide loading. (**D**) Schematic of a passive cavitation detection setup using a 10 MHz transducer quadrature positioned to a focused ultrasound transducer. The broadband signal of cavitation was demonstrated with the Definity positive control. However, there was no cavitation in the PBS, and the only reflector cavitation was present in the K562 suspension. The group with only air bubbles showed a positive correlation between the cavitation energy and the cell destruction fraction, and exhibited significant thermal cavitation. (**E**) Waveforms in the K562 sample were induced by a 100 ms ultrasonic transducer with a reflector to form cavitation bubbles. The pipette bulb was pressurized to 400 kPa to create a pressure chamber. Under the overpressure of thermal cavitation, cell division was inhibited. Significant cavitation (compared to “No US”, ** *p* < 0.01, and *** *p* < 0.001) observed with definity. Adapted with permission from Refs. [114,117,118,119]. Copyright 2020 Future Medicine Ltd., 2017 Elsevier, 2021 American Chemical Society, and 2020 AIP Publishing.

PTX inhibits cancerous cell growth by affecting the microtubules, which depolymerize in the metaphase-to-anaphase transition, so the cell cycle is slowed and dies. PTX is often associated with gastrointestinal and blood-system side effects [114]. By encapsulating PTX within a nanobubble, it may be possible to develop many cancer treatments with fewer side effects (Figure 8B). Additionally, cancer cells are frequently resistant to the use of PTX. Encapsulating the anti-cell death gene si-BCL2 in hepatocellular carcinoma could finally effectively produce a synergistic tumor suppressor effect with PTX. By incorporating specific inhibitors, such as AMD070 (CXCR4 antagonist), PTX can enhance its ability to inhibit tumor proliferation [81]. As of now, nanobubbles can be produced in many different ways to encapsulate PTX, including Herceptin-decorated and ultrasmall superparamagnetic iron oxide [115], polylactic acid/lecithin [116], liposome [91], and poly(lactide-co-glycolide).

#### 5.1.2. Doxorubicin

A major mechanism by which doxorubicin (DOX) affects cancer cells is combining with deoxyribonucleic acid (DNA) and eliminating the DNA-topoisomerase II complex; therefore, it is one of the drugs used in cancer treatment [117]. Unfortunately, DOX has significant adverse effects on normal cells and is inconvenient for cancer patients. In order for DOX to effectively inhibit only the growth of cancer cells, many studies have investigated the use of nanobubbles as encapsulators (Figure 8A). Even though DOX is not easily released when encapsulated in nanobubbles, it can also reduce the toxicity to normal cells and be effective in inhibiting tumor growth at specific locations. Currently available packaging materials include poly(d,l-lactide-co-glycolide)-methoxy-poly(ethylene glycol), superhydrophobic mesoporous silica [120], lipid shell-stabilized perfluoropropane (C_3_F_8_) gas [121], and glycine/poly(ethylene glycol) (PEG)/RGD-modified poly(methacrylic acid), which are able to improve ultrasonic imaging by 1.47 times. A relationship has been found between cancer-associated fibroblasts (CAF) and the development of drug resistance in cancer cells. According to this study, DOX-containing nanobubbles guided by FH peptides were more effective at targeting prostate cancer than DOX alone [122]. Similarly, DOX–nanobubble complex conjugated with nucleolin for targeting triple-negative breast cancer cells has reduced cardiac side effects due to DOX [88]. Similarly, DOX nanobubbles encapsulating siRNA-targeting c-myc also enhanced the ability to kill cancer cells [123].

#### 5.1.3. Temozolomide

Presently, temozolomide (TMZ) is the primary oral drug used to treat high-grade malignant gliomas. It has lipophilic properties and is capable of crossing the blood–brain barrier (Figure 8C). It is a carrier for the transportation of TMZ, covalently conjugated with AS1411, a specific aptamer designed to target glioblastoma (GBM) cells expressing nucleolin. By using ultrasound as a trigger for drug release, the results indicate that nanobubbles can be used to inhibit the growth of GBMs. Furthermore, it should be noted that the compound dramatically enhances the ability of TMZ to suppress tumors [118].

**Table 2:** Ultrasonic therapies for cancer treatment with three different FDA-approved drug delivery systems.

FDA-Approved Drugs	Delivery Vehicles	Cancer Types	Model	Ref.
Paclitaxel	Microbubbles	Breast cancer	In vitro/in vivo	[45]
Paclitaxel	Microbubbles	Cervical cancer	In vitro/in vivo	[124]
Paclitaxel	Microbubbles	Breast cancer	In vitro	[125]
Paclitaxel	Microbubbles	Breast cancer	In vivo	[126]
Paclitaxel	Microbubbles	Pancreatic cancer	In vitro	[127]
Paclitaxel	Microbubbles	Breast cancer	In vitro	[128]
Paclitaxel	Microbubbles	Ovarian cancer	In vitro	[129]
Paclitaxel	Microbubbles	Prostate cancer	In vitro/in vivo	[130]
Paclitaxel	Microbubbles	Endometrium	In vitro	[131]
Paclitaxel	Nanobubbles	Lung cancer	In vitro	[132]
Paclitaxel	Nanobubbles	Lung cancer	In vitro/in vivo	[133]
Paclitaxel	Nanobubbles	Breast cancer	In vitro	[134]
Paclitaxel	Nanobubbles	Breast cancer	In vitro/in vivo	[81]
Paclitaxel	Nanobubbles	Ovarian cancer	In vivo	[106]
Paclitaxel	Nanobubbles	Lung cancer	In vitro	[105]
Paclitaxel	Nanobubbles	Prostate cancer	In vivo	[85]
Paclitaxel/Doxorubicin	Microbubbles	Breast cancer	In vitro/in vivo	[135]
Paclitaxel	Nanobubbles/liposomes	Pancreatic cancer, breast cancer, head and neck cancer	In vitro	[91]
Doxorubicin	Microbubbles	Breast cancer	In vivo	[33]
Doxorubicin	Microbubbles	Glioma	In vitro/in vivo	[73]
Doxorubicin	Microbubbles	Liver cancer	In vitro/in vivo	[136]
Doxorubicin	Microbubbles	Pancreatic cancer	In vitro/in vivo	[137]
Doxorubicin	Microbubbles	Glioblastoma	In vitro/in vivo	[138]
Doxorubicin	Microbubbles	Breast cancer	In vitro/in vivo	[135]
Doxorubicin	Microbubbles	Breast cancer	In vitro/in vivo	[139]
Doxorubicin	Microbubbles	Breast cancer	In vitro/in vivo	[140]
Doxorubicin	Microbubbles	Breast cancer and lung cancer	In vitro/in vivo	[141]
Doxorubicin	Microbubbles	Prostate cancer	In vitro/in vivo	[51]
Doxorubicin	Microbubbles	Breast cancer	In vitro/in vivo	[53]
Doxorubicin	Microbubbles	Pancreatic cancer	In vitro/in vivo	[142]
Doxorubicin	Microbubbles	Breast cancer	In vitro/in vivo	[143]
Doxorubicin	Microbubbles	Colon cancer	In vitro	[144]
Doxorubicin	Microbubbles	Pancreatic cancer	In vitro/in vivo	[145]
Doxorubicin	Microbubbles	Breast cancer	In vitro	[146]
Doxorubicin	Microbubbles	Breast cancer	In vitro	[147]
Doxorubicin	Microbubbles	Liver cancer	In vitro/in vivo	[148]
Doxorubicin	Microbubbles	Melanoma	In vitro/in vivo	[149]
Doxorubicin	Microbubbles	Breast cancer and lung cancer	In vitro	[150]
Doxorubicin	Microbubbles	Liver cancer	In vivo	[57]
Doxorubicin	Microbubbles	Liver cancer	In vitro/in vivo	[151]
Doxorubicin	Microbubbles	Liver cancer	In vitro/in vivo	[61]
Doxorubicin	Microbubbles	Liver cancer	In vitro/in vivo	[152]
Doxorubicin	Microbubbles	Breast cancer	In vitro	[153]
Doxorubicin	Microbubbles	Pancreatic cancer	In vitro/in vivo	[67]
Doxorubicin	Microbubbles	Breast cancer	In vitro/in vivo	[154]
Doxorubicin	Microbubbles	Bladder cancer	In vivo	[155]
Doxorubicin	Microbubbles	Breast cancer	In vitro	[156]
Doxorubicin	Nanobubbles	Breast cancer	In vitro	[157]
Doxorubicin	Nanobubbles	Colon cancer	In vitro/in vivo	[158]
Doxorubicin	Nanobubbles	Breast cancer	In vitro/in vivo	[141]
Doxorubicin	Nanobubbles	Breast cancer and cervical cancer	In vitro	[159]
Doxorubicin	Nanobubbles	Ovarian cancer	In vitro/in vivo	[121]
Doxorubicin	Nanobubbles	Breast cancer	In vitro/in vivo	[86]
Doxorubicin	Nanobubbles	Breast cancer	In vitro/in vivo	[88]
Temozolomide	Nanobubbles	Glioblastoma	In vitro/in vivo	[160]
Temozolomide	Liposomes	Glioblastoma	In vitro/in vivo	[161]
Temozolomide	Liposomes	Glioblastoma	In vitro/in vivo	[162]

### 5.2. Ultrasound-Focused Pain Relief and Local Tumor Control

In pain control, pain from cancer is the most difficult to manage. In particular, the pain caused by bone metastases can be bone-eroding, and it is difficult to obtain rapid and effective relief with a single treatment [119]. Magnetic resonance-guided focused ultrasound (MRgFUS) provides a new option for noninvasive pain management (Figure 8D). The principle of MRgFUS treatment is to use the MRI navigation system to accurately locate and focus the ultrasound on the area of bone pain, heat it to above 60 °C, and use thermal ablation to destroy the nerve tissue and target the tumor on the surface of the bone that is causing the pain (Figure 8E). At the same time, the surrounding normal tissue is preserved to achieve the therapeutic purpose of pain relief and local tumor control. MRgFUS is suitable for patients with bone metastases for whom palliative radiation therapy is ineffective and for those with bone metastases who are unwilling to receive radiation therapy. MRgFUS adopts a single treatment method with a short recovery period and few complications. A small number of patients may experience pain, skin burns, nerve damage, deep vein thrombosis, or contrast agent allergy during the treatment process. In a multinational, multicenter study, the mean pain intensity score (10 cm-VAS) decreased from 5.9 to 3.8 at 3 days after treatment. In the third month after treatment, the mean pain intensity decreased to 1.8, and bone mineral density was found in the treatment area. Overall, this new treatment effectively manages pain from bone metastases, improving patient quality of life.

### 5.3. Synergistic Treatment of Ultrasound and Nanomaterials

Nanoparticle-assisted ultrasound therapy (NAUT) is a new type of tumor treatment. A study found that ultrasound kills cancer cells by adding nanoparticles to a petri dish, culturing the cells together, and then irradiating them with a medium-energy ultrasound. Normal cell function was preserved. Such a method is expected to reduce the side effects caused by traditional cancer treatments effectively. However, the mechanism behind its selective targeting of cancer cells remains unclear. Previous studies have hypothesized whether nanoparticles can be used as nucleation sites to reduce the threshold of the cavitation effect, thereby increasing the cavitation effect [160]. The Weissler reaction was used to quantify the cavitation effect and to investigate whether adding different amounts of nanoparticles to a solution could increase the cavitation effect. At the beginning of the experiment, gold nanoparticles were used as nanomaterials. However, they found that the potassium iodide reagent had limitations and would react with the added gold nanoparticles, interfering with the experimental results. Then, the correlation between the nano-polystyrene particles and the cavitation effect was observed [163]. There was no difference in the amount of triiodide ion signal recorded between the group containing nano-polystyrene and the group without nanoparticles [164]. One significant difference is that the addition of nanoparticles cannot directly increase the generation of cavitation effects. The usefulness of NAUT treatment may be due to ultrasonic thermal or other mechanical effects rather than cavitation; thus, the actual mechanism needs further investigation.

Recently, combining inorganic nanomaterials with ultrasonic contrast agents to achieve the NAUT process has come into vogue. Chan et al. studied the combination of upconversion nanoparticles and graphical carbon nitride quantum dots and embedded them in nanobubbles, using the ultrasonic imaging of nanobubbles and the fluorescence imaging of upconversion nanoparticles in order to employ multiple imaging [165]. In this study, fluorescence and ultrasound imaging were combined to achieve various imaging effects, solving the problems of insufficient fluorescence penetration depth and the poor resolution of the ultrasound. Based on the research described above, Cheng et al. used nanobubbles that could be subjected to ultrasonic shock to generate cavitation and encapsulated drugs, and they combined these with long-afterglow nanoparticles [118]. They delivered them to the brain for brain tumor treatment and tracking. After the composite material was ultrasonically treated, the bubbles produced a cavitation effect because the internal gas medium was different from the liquid environment, temporarily opening the blood–brain barrier and sending temozolomide and near-infrared luminescent long-afterglow nanoparticles into the brain for medical treatment.

## 6. Conclusions

In recent years, many studies have successfully prepared a variety of multifunctional, nano-sized micelles and bubbles, which can be used for magnetic resonance imaging, ultrasonography, carrying hydrophobic anticancer drugs, and improving drug delivery efficiency. This review summarizes several commonly used ultrasound contrast agents that can be used to increase the accuracy of treatment positioning and enhance the treatment effect. However, it still faces many challenges, even with contrast agents, to improve the ultrasound diagnostic. For instance, (1) the use of contrast agents is easily obstructed by air: air can block the ultrasound, and if the patient’s stomach becomes severely inflated, the organ’s structure cannot be seen with ultrasound; (2) the structure behind the bone cannot be seen: the bone will reflect the ultrasound, so the ultrasound cannot see the structure behind the bone; (3) severe obesity may affect the accuracy: in some overly obese patients, ultrasound cannot reach the organs deeply, which will also cause problems in the examination; (4) limited resolution: despite the use of contrast agents, the resolution of ultrasound is still limited due to the poor stability of the contrast agent itself, and the structure will disintegrate when high-frequency ultrasound is used, meaning it can no longer enhance the contrast. Based on these limitations, magnetic navigation ultrasound has often been used to assist positioning in recent years, improving shortcomings and finding the correct position when using ultrasound images. The image can be uploaded to the ultrasound machine first. Through the synchronous positioning system, with the guidance of computed tomography or nuclear magnetic resonance, the relative location of tumor tissue can be found under ultrasound, and the success rate of cancer treatment can be significantly improved.
